# Clinical Study of Critical Patients with Hemorrhagic Fever with Renal Syndrome Complicated by Acute Respiratory Distress Syndrome

**DOI:** 10.1371/journal.pone.0089740

**Published:** 2014-02-24

**Authors:** Hong Du, Jing Li, Wei Jiang, Haitao Yu, Ye Zhang, Junning Wang, Pingzhong Wang, Xuefan Bai

**Affiliations:** Center for Infectious Diseases, Tangdu Hospital, Fourth Military Medical University, Xi’an, Shaanxi, China; University of Rochester, United States of America

## Abstract

**Objectives:**

The aim of this study was to investigate the clinical characteristics and outcomes of critical patients with hemorrhagic fever with renal syndrome (HFRS) complicated by acute respiratory distress syndrome (ARDS).

**Materials and Methods:**

To observe the demographic, epidemiological and clinical characteristics, and to explore the predictive effects for prognosis in laboratory findings, we conducted a detailed retrospective analysis of clinical records for critical patients with HFRS complicated by ARDS, treated at the center for infectious diseases, Tangdu Hospital, between January 2008 and December 2012.

**Results:**

A total of 48 critical patients with laboratory confirmed HFRS accompanied by ARDS were enrolled in the study, including 27 survivors and 21 non-survivors, with a fatality rate of 43.75%. Thirty-one individuals (64.6%) contracted HFRS between the months of September and December. The non-survivors tended to have lower incidence of overlapping phase (P = 0.025). There were no obvious differences in the needs for mechanical ventilation (MV) and renal replacement therapy (RRT), except for the need for vasoactive drugs between the survivors and non-survivors (P = 0.001). The non-survivors were found to have higher frequencies of encephalopathy, refractory shock and multiple organ dysfunction syndrome (MODS), lower incidences of acute renal failure (ARF) and secondary hypertension (P<0.05). The non-survivors tended to have lower levels of serum creatinine (Scr) (P<0.001) and fibrinogen (Fib) (P = 0.003), higher incidences of prolonged prothrombin time (PT) (P = 0.006) and activated partial thromboplastin time (APTT) (P = 0.020) and higher levels of aspartate aminotransferase (AST) (P = 0.015), and the laboratory parameters mentioned above reached statistical significance for predicting prognosis (P<0.05).

**Conclusion:**

The high mortality rate of critical patients with HFRS complicated by ARDS emphasizes the importance of clinicians’ alertness and timely initiation of systemic supportive therapy.

## Introduction

Hantaviruses are enveloped RNA viruses of the family *Bunyaviridae* [1]. These emerging zoonotic pathogens cause two distinct syndromes in humans: hemorrhagic fever with renal syndrome (HFRS) in Europe and Asia and hantavirus pulmonary syndrome (HPS) in the Americas [2], [3]. China is the most severe endemic area of HFRS in the world, with 30,000–50,000 cases reported annually, which account for > 90% of the total number of cases worldwide [4], [5]. The Shaanxi province is one of the most severely affected provinces in China, and Xi’an city is the central district of the Shaanxi province, with an increasing incidence and mortality rate in the last three years [6].

A hallmark of HFRS is capillary leak syndrome, causing edema and hemorrhage, which suggests that the vascular endothelium may be the prime target of the virus infection [7], [8], [9].Typical cases of HFRS progress through five successive phases: febrile, hypotensive, oliguric, diuretic and convalescent [10], [11], [12]. In some grave cases, the febrile, hypotensive and oliguric phases can overlap, resulting in acute progressive noncardiogenic pulmonary edema, appearing as acute respiratory distress syndrome (ARDS) and leading to greatly increased fatality rate. As far as we know, ARDS has become a major cause of death on severe sepsis patients treated in the intensive care unit (ICU) [13].

In the last five years, more than 1,200 cases of symptomatic patients with laboratory confirmed HFRS were treated at the Center for Infectious Diseases, Tangdu Hospital, a 300-bed primary care and tertiary referral medical center in the northeast district of China. In view of the higher incidence and hospital mortality rate of critical patients with HFRS complicated by ARDS, it is essential to gain a better, more comprehensive understanding of the clinical characteristics and outcomes of the patients, and to help clinicians provide timely monitoring and effective supportive therapy during the early stage of this disease.

## Materials and Methods

### Ethics Statement

This retrospective study was reviewed and approved by the Institutional Review Board of Tangdu Hospital. The patients’ medical records were anonymized and de-identified prior to analysis.

### Study Participants

The records of 432 typical patients with laboratory confirmed HFRS, treated at the Center for Infectious Diseases, Tangdu Hospital, between January 2008 and December 2012 were reviewed. The medical records of the selected patients were reviewed for analysis of the demographic, epidemiologic and clinical conditions, complications, outcomes and major supportive therapies. Patients who had other kidney diseases, diabetes, cardiovascular disease, hematological disease, autoimmune disease, viral hepatitis, and other liver diseases were excluded from this study.

Based upon the degree of hypotension, renal function, effusion, hemorrhage, and edema of the patients, the severity of HFRS was classified into four types [14]: (1) mild, defined as patients who had kidney injury without oliguria and hypotension; (2) moderate, defined as patients who had uremia, effusion (bulbar conjunctiva), hypotension, hemorrhage (skin and mucous membranes), and AKI with typical oliguria; (3) severe, defined as patients who had severe uremia, effusion (bulbar conjunctiva and either peritoneum or pleura), hemorrhage (skin and mucous membranes), hypotension and AKI with oliguria (urine output of 50–500 mL/day) for ≤5 days or anuria (urine output of <100 mL/day) for ≤2 days;(4) critical, defined as patients who usually had one or more of the following complications compared with the severe patients: refractory shock (≥2 days), visceral hemorrhage, heart failure, pulmonary edema, brain edema, severe secondary infection, and severe AKI with oliguria (urine output of 50–500 mL/day) for >5 days or anuria (urine output of <100 mL/day) for >2 days. Commonly, the so-called acute stage of the disease is defined as the period of febrile, hypotensive and oliguric phases; overlapping phases are defined as periods with overlapping febrile, hypotensive and oliguric phase. Overall, eighty-six of the cases were classified as of the critical type. According to the Berlin Definition of ARDS [15], forty-eight of the 86 critical patients exhibited ARDS during the clinical course and were eventually enrolled in this study.

### Laboratory and Imaging Studies

Laboratory and imaging results that documented the clinical presentations and outcomes of the critical patients with HFRS complicated by ARDS were analyzed and compared. Biochemical tests of blood samples were performed using an autoanalyzer (Sysmex, XT-4000i, Japan), including basic metabolic,liver and renal function and glucose tests. Blood clotting functions were tested using hematology analyzers (CA7000, Sysmex, Japan; ACL, TOP700, United States). Chest and abdomen organs were visualized using X-ray radiography (PLOYMOBIL 2.5, Siemens, Germany) and ultrasonography (DC-6, MINDRAY, China). Computed tomography (CT) (CTTM64, Siemens, Germany) was performed in some patients. Cardiac function was measured using Cardiofax (1350p, NIHON KOHDEN, Japan) and ultrasonography (DC-7, MINDRAY, China). Arterial blood gases were measured using an automatic blood gas system (ABL80, Denmark). Hemo cultures were tested using an autoanalyzer (BD9120, United States).

The diagnosis of HFRS was made based upon the detection of specific IgM and IgG antibodies to Hantaan virus (HTNV) in acute phase serum specimens by enzyme-linked immunosorbent (ELISA) assay. The assay was performed using IgG/IgM capture ELISA kits, which were analyzed using a multifunctional autoanalyzer(BIORAD-680, United States).

### Renal replacement therapy (RRT)

There were two major patterns of RRT used in the study patients in our center: continuous renal replacement therapy (CRRT) and intermittent hematodialysis (IHD). According to the Chinese Medical Association’s guide for blood purification in the ICU [16], critical patients with evidence of unstable hemodynamic status accompanied by multiple organ injury, pulmonary edema, fluid overload,severe electrolyte disturbances,encephalopathy and AKI were started with CRRT. For patients with stable hemodynamic status and less potentially fatal complications during their clinical course, IHD was chosen by clinicians as the first choice. However, over the period of treatment, a minority of critical patients may have lost the option of CRRT because of their critical condition on admission, and some of them even signed informed consents to withdraw RRT due to poor economic status.

Patients treated with CRRT were catheterized in the femoral vein with a double-lumen catheter. Replacement of catheters was performed in some patients with critical condition. The median duration of catheterization was 12 days (range of 3–28 days), and AN69 Hemofilters were used for patients treated with a PRISMA machine with an M100 extra-corporeal circuit. Substitution fluid: port prescription was used in all patients, and a bicarbonate buffer solution and other fluids were added separately. CRRT (24 h) was provided for most patients during the acute stage, particularly during the hypotensive overlapping oliguric phase. The main modality of CRRT was continuous venovenous hemodiafiltration (CVVHDF). Fluids were exchanged at a rate of 25–35 ml/(kg·h). To prevent coagulation, the filter and circuit were primed with heparinized saline. Heparin was given at an initial dose of 5–10 iu/kg and was maintained at a dose of 5–10 iu/kg/h in patients without bleeding tendency. No anticoagulant was used in patients with bleeding tendency, and frequent washing of the filter with substitution fluid was performed.

Patients treated with IHD were catheterized in the femoral vein with a double-lumen catheter. Ployflux 17L Capillary Dialyzers were used on patients treated with a hemodialyzer (AK96S, Gambro, Sweden). The dialysate flow rate was 400–600 ml/min with bagged fluid. All patients were treated with bicarbonate dialysate at a sodium concentration of 140 mmol/L. All patients were heparinized with low dose heparin or administered no anticoagulant and dialyzed for 3.0–4.0 hours, depending on the patient’s condition.

### Mechanical ventilation

According to the guidelines for the diagnosis and treatment of ALI/ARDS [13], the majority of the critical patients were treated with invasive mechanical ventilation (MV) (PB760, Tyco, United States) or noninvasive MV (SMARTAIR ST, AIROX, France) during the acute stage. However, for a minority of the critical patients, who eventually died because of their critical conditions or apparent respiratory and cardiac arrest on admission, family members provided informed consents to withdraw any invasive supporting treatments, including MV and hemodynamic monitoring due to poor economic status.

### Definition of HFRS-related complications

Acute renal failure (ARF) was defined as the third stage of AKI according to the criteria of the Acute Kidney Injury Network (AKIN) [17]. Patients who manifested altered mental status (drowsiness, spasm, lethargy, agitation, or coma) were defined as having encephalopathy. Patients who manifested acute respiratory distress within a duration of one week including dyspnea, shortness of breath, cyanosis, pleural effusions on X-ray, and hypoxemia accompanied by oxygenation index (PaO_2_/FiO_2_) ≤200 mmHg that could not be explained by cardiac dysfunction and fluid overload were defined as having ARDS [15]. Gastrointestinal hemorrhage was defined as hematemesis or dark stools with hemodynamic instability and rapid decline of hemoglobin level to ≤7.0 g/dL. Secondary hyperglycemia was defined as an increase of fasting blood sugar ≥7.1 mmol/L without primary diabetes. Concurrent bacteremia was defined as a positive bacterial growth from blood culture within 72 h of hospital admission. Secondary hypertension was defined as systolic blood pressure (SBP) ≥140 mmHg during the hospital course without primary hypertension. Arrhythmia, including atrial premature beat, ventricular premature contraction, atrial fibrillation, ventricular fibrillation, supraventricular tachycardia, ventricular tachycardia and atrial ventricular block was confirmed by electrocardiography (ECG). Refractory shock was defined as shock of ≥ 24 h duration, which could not be resolved by fluid resuscitation or vasoactive drugs. In this study, MODS was defined as organ dysfunction involving two or more organs, with the exception of the lungs, during the acute stage after hospitalization, according to the current definition of MODS [18], [19]. Patients who had upper gastrointestinal tract symptoms, including abdominal distension,nausea, vomiting and upper abdominal pain accompanied by elevated blood and urinary amylase and adrenomegaly on imaging were defined as having acute pancreatitis (AP) [20], [21].

### Statistical Analyses

Statistical analyses were performed using the SPSS 17.0 software (SPSS Inc, Chicago, IL, USA). The tables were prepared using Excel 2003 (Microsoft), and the figure was drawn using GraphPad Prism 5 (GraphPad Software, SanDiego CA). The demographic, clinical, laboratorial and imaging data between the survivors and non-survivors were compared using univariate analysis. Continuous variables were presented as means ± SD and analyzed using the Kolmogorov-Smirnov test for distribution normality and Levene’s test for the homogeneity of variance. For normally distributed variables, the data were compared using Student’s t-test. For variables with non-Gaussian distribution, the nonparametric Mann-Whitney U-test was used. The frequencies and percentages of qualitative variables were calculated. Significant differences between the survivors and non-survivors were tested by the chi-square test, and Fisher’s exact test was used when numbers were too small to perform the chi-square test. Spearman’s correlation coefficient was used to determine the correlation between laboratory values, complications and survival outcomes. Predictive value for the prognosis of the laboratory results were tested using receiver operating characteristic (ROC) curves and calculated by measuring the area under the curve (AUC) and 95% confidence interval (CI). A two-tailed P < 0.05 was considered statistically significant.

## Results

### Clinical, demographic and epidemiologic characteristics of the critical patients with HFRS complicated by ARDS

Of the 48 critical patients with laboratory confirmed HFRS accompanied by ARDS, 27 patients survived and 21 died, with a fatality rate of 43.75%. Of the 21 non-survivors, 16 cases died during the overlapping phase within the first week from the onset of the illness. 38 patients (79.2%) were male, and 35 cases (72.9%) reported ‘farmer’ as their occupation. The mean age and sex of the survivors and non-survivors were not significantly different (P = 0.078 and P = 0.929, respectively). There were also no significant differences in the seasonal incidence (September to December), occupation, interval from symptom onset to patients’ hospital arrival and duration of febrile phase (P = 0.732, P = 0.653, P = 0.936, P = 0.385, respectively) (Table 1). The incidence of the overlapping phase in the non-survivors was greater than that of the survivors (P = 0.025) (Table 1). Of the main supportive treatments, there were no significant differences in the frequencies of MV and RRT, except for the need for vasoactive drugs (P = 0.001) (Table 1).

**Table 1:** Clinical, epidemiological and demographic characteristics of critical patients with HFRS complicated by ARDS

Variables	Survivors (n = 27)	Non-survivors (n = 21)	p value*
Mean age, years	46.41±13.36	52.71±11.82	0.078
Male/Female, n	22/5	16/5	0.929
Occupation (farmer), n (%)	19 (70.4)	16 (76.2)	0.653
Seasonal incidence (September to December), n (%)	18 (66.7)	13 (62.9)	0.732
Frequency of MV (n/%), n (%)	24 (88.9)	18 (85.7)	1.000
Single invasive MV, n (%)	18 (66.7)	13 (62.9)	0.732
Single noninvasive MV, n (%)	3 (11.1)	2 (9.5)	1.000
Combination, n (%)	3 (11.1)	3 (14.3)	1.000
Incidence of overlapping phase, n (%)	15 (55.6)	18 (85.7)	0.025
Frequency of RRT, n (%)	26 (95.3)	16 (76.2)	0.099
CRRT initiated, n (%)	18 (66.7)	13 (61.9)	0.732
Frequency of vasoactive drugs, n (%)	8 (29.6)	16 (76.2)	0.001
Interval from onset to admission, days	4.04±1.72	4.00±1.34	0.936
Duration of febrile phase, days	5.04±1.87	4.62±1.43	0.385

Mean ± SD for continuous variables, n (%) for categorical variables.

MV, mechanical ventilation; RRT, renal replacement therapy; CRRT, continuous renal replacement therapy.

* Survivors vs. non-survivors.

### Complication related to HFRS in critical patients with ARDS

Among HFRS-related complications, the incidences of encephalopathy, refractory shock and MODS in the non-survivors were significantly higher than that of the survivors(P<0.001), while the incidences of ARF and secondary hypertension in the survivors were significantly higher than that of the non-survivors (P = 0.011 and P = 0.003, respectively) (Table 2). No significant differences in the frequencies of arrhythmia, gastrointestinal hemorrhage, hyperglycemia, concurrent bacteremia and acute pancreatitis were identified (P>0.05) (Table 2).

**Table 2:** HFRS-related complications in critical patients with ARDS.

Variables	Survivors (n = 27)	Non-survivors (n = 21)	p value*
Arrhythmia, n (%)	6 (22.2)	4 (19.0)	1.000
Gastrointestinal hemorrhage, n (%)	10 (37.0)	12 (57.1)	0.165
Hyperglycemia, n (%)	22 (81.5)	18 (85.7)	1.000
Encephalopathy, n (%)	6 (22.2)	20 (95.2)	<0.001
Concurrent bacteremia, n (%)	11 (40.7)	5 (23.8)	0.217
Secondary hypertension, n (%)	18 (66.7)	5 (23.8)	0.003
Refractory shock, n (%)	2 (7.4)	13 (61.9)	<0.001
MODS, n (%)	4 (14.8)	13 (61.9)	0.001
AP, n (%)	8 (29.6)	3 (14.3)	0.364
ARF, n (%)	25 (92.6)	12 (57.1)	0.011

n (%) for categorical variables.

MODS, multiple organ dysfunction syndrome; AP, acute pancreatitis; ARF, acute renal failure.

* Survivors vs. non-survivors.

### Laboratory findings in critical patients with HFRS complicated by ARDS during the acute stage

Of the critical study patients, the mean levels of the maximum leukocyte counts, alanine aminotransferase (ALT), aspartate aminotransferase (AST), serum creatinine (Scr) and glucose during the acute stage were higher than the reference values; the prothrombin time (PT), activated partial thromboplastin time (APTT) and thrombin time (TT) during the acute stage were more prolonged than the reference values. Except for the levels of the maximum mahemoglobin (HGB) and uric acid (UA) in the non-survivors; the means levels of the nadir platelet (PLT), serum albumin (ALB) and minimum fibrinogen (Fib) were lower or shorter than the reference values (P<0.001) (Table 3). Compared with the survivors, the non-survivors were found to have lower Scr (P<0.001) and Fib (P = 0.003), and to have higher incidences of prolonged PT (P = 0.006), and APTT (P = 0.020), as well as higher levels of AST (P = 0.015) (Table 3).

**Table 3:** Laboratory findings in critical patients with HFRS complicated by ARDS during the acute stage.

Variables	Survivors (n = 27)	Non-survivors (n = 21)	p value•
Maximum leukocyte counts, ×10^9^/L	35.95±15.49*	46.93±22.45*	0.051
Nadir PLT, ×10^9^/L	11.81±12.93**	15.29±10.94**	0.330
Maximum HGB, g/L	170.96±22.29*	162.48±19.94	0.178
Maximum ALT, U/L	208.07±256.04*	350.33±324.42*	0.096
Nadir serum ALB, g/L	22.74±5.14**	21.31±5.16**	0.344
Maximum Scr, µmol/L	788.95±236.27*	411.88±197.49*	<0.001
Maximum UA, µmol/L	543.20±213.00*	433.76±154.57	0.054
Maximum glucose, mmol/L	13.58±6.30*	14.52±8.30*	0.656
Longest PT, sec	15.95±3.87*	23.73±11.32*	0.006
Longest APTT, sec	50.92±16.16*	63.58±20.13*	0.020
Longest TT, sec	25.19±6.79*	26.44±7.49*	0.554
Minimum Fib, g/L	1.63±0.64**	1.13±0.40**	0.003
Maximum AST, U/L	370.33±406.15*	1271.9±1531.14*	0.015

Reference values: Leukocyte count (3.2–9.7)×109/L; PLT (100–300)×109/L; HGB (120–160) g/L (male), (110–150) g/L (female); ALT (4–44) U/L; AST (8–38) U/L; serum ALB (35–55) g/L; serum Cr (53–97) µmol/L (male), (35–71) µmol/L (female); UA (210–430) µmol/L (male), (150–360) µmol/L (female); glucose (3.89–6.11) mmol/L; PT (8.8–13.8) sec; APTT (25.1–36.5) sec; TT (10.3–16.6) sec, and Fib (2.38–4.98) g/L.

PLT, platelet; HGB, hemoglobin; ALT, alanine aminotransferase; ALB, albumin; Scr, serum creatinine; UA, uric acid; PT, prothrombin time; APTT, activated partial thromboplastin time; TT, thrombin time; Fib, fibrinogen;AST, aspartate aminotransferase.

• Survivors vs. non-survivors.

* Higher or longer than the reference value, P<0.05.

** Lower than the reference value, P<0.05.

### Spearman correlation and ROC analysis

Of the laboratory data, Spearman correlation analysis revealed that the levels of AST, PT and APTT were negatively correlated with survival outcome, while Scr and Fib were positively correlated with survival outcome (P<0.05) (Table 4).

**Table 4:** Spearman correlation analysis of laboratory data and patient outcome.

Variables	Outcome		Variables	Outcome	
	r	p value		r	p value
Scr	0.650	<0.001	Fib	0.420	0.003
PT	–0.416	0.003	AST	–0.453	0.001
APTT	–0.327	0.023			

r, correlation coefficient; Scr, serum creatinine; PT, prothrombin time; APTT, activated partial thromboplastin time; Fib, fibrinogen;AST, aspartate aminotransferase.

**Figure 1:**
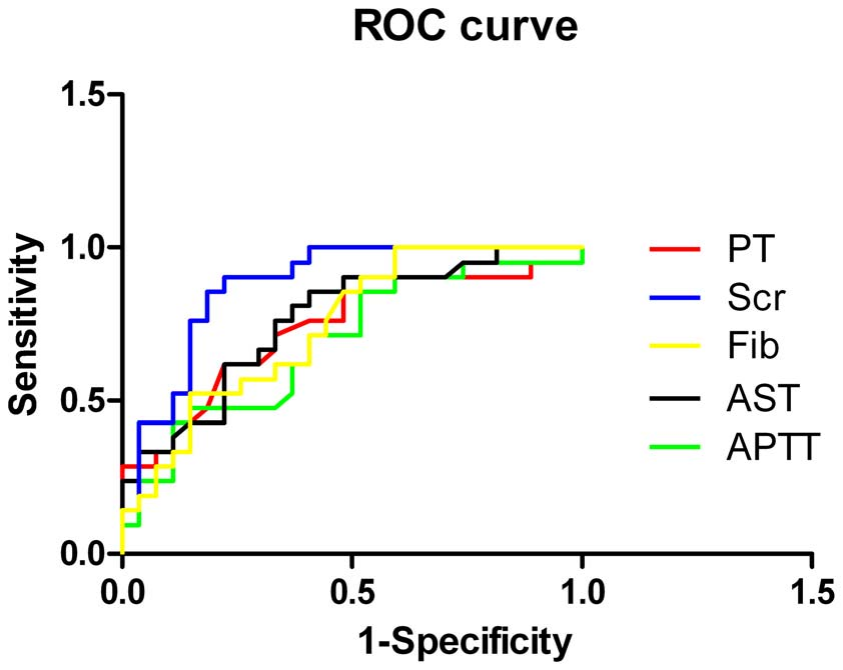
ROC analysis of laboratory data in predicting death in critical patients with HFRS complicated by ARDS.

To explore the predictive value of the laboratory data for prognosis, the ROC and AUC were analyzed. The results for Scr, Fib, AST, PT and APTT were all found to be statistically significant for predicting prognosis (P<0.05), with AUCs of 0.878, 0.744, 0.764, 0.742 and 0.690, respectively (Figure 1,Table 5).

**Table 5:** Predictive values for prognosis of laboratory data in critical patients with HFRS accompanied by ARDS.

Variables	AUC	P value^a^	Cut-off value^b^	Sensitivity^c^	Specificity^c^	95% CI^c^	
						Lower	Upper
Scr^c^	0.878	<0.001	660.85	90.5	77.8	78.1	97.6
PT	0.742	0.004	18.15	61.9	77.8	59.6	88.7
APTT	0.690	0.025	43.2	85.7	48.1	53.8	84.3
			66.6	47.6	85.2		
Fib^d^	0.744	0.004	1.106	52.4	85.2	60.7	88.1
			1.429	85.7	51.9		
AST	0.764	0.002	310	76.2	66.7	62.9	89.9

AUC, area under the curve; CI, confidence interval.

a P value for calculated AUC in predicting death (ROC analysis).

b Units of measure of the cut-off values are µmol/L, sec, sec, g/L and U/L, respectively.

c Sensitivity, specificity and 95% confidence interval are presented as percentages.

d Test direction: lower test result indicates a more positive test.

## Discussion

As far as we know, this is the first clinical investigation of critical patients with HFRS complicated by ARDS in China. In this study, we compared the demographic, epidemiological, clinical and laboratory characteristics of the survivor and non-survivors, and identified the predictive value of laboratory finding on patients’ survival.

In this study, forty-eight of the 86 critical patients with HFRS reviewed exhibited ARDS during the acute stage with an incidence of 55.81%, which indicates that ARDS is a common clinical complication in critical patients infected by HTNV in Xi’an city [6]. Of the 48 critical patients with HFRS enrolled, 21 patients died, representing a high fatality rate of 43.75%, which was similar with that of patients with severe septic or septic shock in the ICU [22], [23]. The pathophysiologic mechanism of ARDS is thought to involve continuous fluid leakage from the intravascular lumen to the extravascular compartment and the lung alveolar space [24], [25]. Analogous to several reports of patients with HFRS infected by Puumala virus (PUUV) in the Europe [26]–[28], this study also revealed that more critical HFRS patients in the northwest region of China can manifest pulmonary-renal syndrome during the clinical course, leading to high permeability pulmonary edema and hypoxic respiratory failure associated with high mortality rate. Not only the hantavirus pulmonary syndrome (HPS) prevailed in the America continent [29], hantavirus infection should also be considered as a cause of acute respiratory distress in all endemic areas worldwide.

HFRS is widely accepted as having the basic clinical characteristics of systemic inflammatory response syndrome (SIRS), and the patient’s pathophysiologic manifestations of the hypotensive phase are similar to those of typical distributive shock [5]. Acute progressive noncardiogenic pulmonary edema is a main cause of ARDS in critical patients with HFRS, which is commonly accompanied by a concurrent marked hemoconcentration during the acute stage, especially the hypotensive phase. Generally, the hypotensive phase of HFRS (e.g., low blood pressure and circulatory collapse) occurs between day 3 and day 7 of the clinical course. Some critical patients even have overlapping phases combined with various fatal complications such as refractory shock, encephalopathy and MODS except for ARDS, which was demonstrated in the present study (Table 2). Our study revealed that the frequency of administration of vasoactive drugs in non-survivors was higher, while the frequencies of MV and RRT were not significantly different between the survivors and non-survivors (Table 1). This result appears to indicate that the invasive supportive treatments mentioned above were not capable of altering the final prognosis. We acknowledge that a minority of patients in the non-survivors were treated conservatively without the use of MV and RRT because of their critical condition on admission and their poor financial means, which may have influenced the statistical significance of the result to some degree. Although no specific antiviral therapy for HFRS is currently available, earlier diagnosis, earlier monitoring, earlier admission to the ICU, and timely and systematically supportive therapy would be necessary to ensure patients’ survival during the acute stage and significantly reduce the mortality rate compared to critical patients with HFRS treated conservatively [4], [30].

Of the 48 critical patients with HFRS complicated by ARDS, 38 patients (79.2%) were male and the occupation of 35 patients (79.2%) was farming. A total of 31 patients (64.6%) contracted the disease between September and December. Until now, the hantavirus strains from host rodents and patients in Xi’an only belong to the HTNV, and SEOV strains have not been found [6]. The high incidence and seasonal distribution (Table 1) are consistent with the known biological and epidemical characteristics of the predominant natural host of HTNV, *Apodemus agrarius*, and the susceptibility of patients working in surroundings that are the natural habitats of rodents in Xi'an city [5], [6]. Furthermore, the incidence of the overlapping phase in non-survivors was higher than that of the survivors (Table 1), which was consistent with the clinical course of critical HFRS. After all, some critical patients died of fatal clinical complications during the acute stage, particularly during the hypotensive phase or overlapping phases. Almost all the critical patients who survived the overlapping phases manifested obvious oliguria with AKI. Because of the reasonable utilization of RRT, including CRRT and IHD [31], only a minority of the patients would have died of AKI.

Kidney is a major organ damaged during HFRS, and previous research had demonstrated that the most prominent pathological presentation was acute tubulointerstitial nephritis following the infiltration of inflammatory cells [32]. In this study, 37 critical patients with HFRS complicated by ARDS (77.1%) developed ARF, the third stage of AKI [17] (Table 2). Interestingly, we observed that the incidence of ARF in the survivors was significantly higher than that of the non-survivors (Table 2). We also observed that Scr levels were positively correlated with the survival outcome by correlation analysis (Table 4). Non-survivors were also found to have lower Scr levels (Table 3), and a low level of Scr was statistically associated with increased mortality, with an AUC of 0.878 (Table 4, Figure 1). As it mentioned above, the overlapping phase usually occurs within the first week of the clinical course, and some critical patients may have died of fatal complications during the phase when ARF was not detected. Once the patients pass through the overlapping phase, severe AKI and secondary hypertension will become the major clinical complications accompanied with the occurrence of single oliguric and diuretic phase. Given that a majority of the critical patients would survive the oliguric and diuretic phase because of the reasonable application of RRT and vasoactive agents, the stage of AKI and secondary hypertension have not been the direct influential factors for prognosis.

In our study, univariate analysis demonstrated that non-survivors had lower levels of Scr and Fib, and higher incidences of prolonged PT, APTT and higher levels of AST (Table 3). Spearman correlation analysis revealed that AST, PT and APTT were negatively correlated with survival outcome, whereas Fib was positively correlated with survivor outcome (Table 4). ROC analysis also revealed that Fib, AST, PT and APTT were all statistically significant for predicting patient prognosis, with AUC of 0.744, 0.764, 0.742 and 0.690, respectively (Figure 1,Table 5). This observation indicated that critical patients with HFRS complicated by ARDS often experienced hepatic, cardiac and kidney injuries. Stress hyperglycemia and hypermetabolism were also common during the acute stage. Severe plasma leakage, massive bleeding and profound shock are known to lead to tissue hypoperfusion, potentially triggering AKI and hepatic injury. Furthermore, the non-survivors appeared to be in more critical condition, with a higher degree of inflammation and coagulation abnormality compared with the survivors. The high concentration of HGB was closely related to the degree of pachyemia; the low level of PLT may reflect decreased platelet production or increased platelet consumption, which may be associated with the severity of the underlying hemorrhage [33], [34]. Furthermore, the low level of ALB correlated with degree of the loss of vascular integrity, enhanced vascular permeability and liver dysfunction [35]. Overall, all the laboratory parameters discussed above reflected the severity of the disease, and clinicians should pay attention to their dynamic changes that occur during the acute stage, by close monitoring and initiation of timely management, as indicated.

Being a retrospective study, some limitations must be addressed. First, our study was conducted at the largest center for infectious diseases in the northwest region of China. Most patients with severe clinical conditions come to our medical center, but a significant number of critical patients may still have been admitted to local hospitals. Therefore, the demographic and epidemiological characteristics and mortality rate of the critical patients with HFRS complicated by ARDS in our study may have been biased. Second, we were unable to calculate the acute physiology, age, chronic health evaluation (APACHE II) score [36], sepsis related organ failure assessment (SOFA) [37] and simplified acute physiology score (SAPS II) [38] of the patients on admission in this study because of the loss of detailed clinical data on central nervous system dysfunction. These data may be very important for predicting the prognosis and the diagnosis of these clinical conditions may have been biased based upon clinicians’ personal recognition of the clinical severity of this disease. Third, the relatively small number of cases limited the statistical power of our study. Finally, the clinical outcomes and classifications of patients with HFRS might be biased due to the lack of a more standardized protocol for the management of patients with HFRS. For example, the choice of resuscitation fluid (i.e., crystal or colloid solution), optimal dose of vasoactive agents, optimal timing and modality of MV, which are each important in the treatment of HFRS, were not standardized in this study.

In summary, the high mortality rate of critical patients with HFRS complicated by ARDS underscores the importance of clinicians’ alertness and timely initiation of systematic supportive therapy.
